# Anthropometric Prediction of DXA-Measured Percentage of Fat Mass in Athletes With Unilateral Lower Limb Amputation

**DOI:** 10.3389/fphys.2020.620040

**Published:** 2020-12-23

**Authors:** Valentina Cavedon, Marco Sandri, Massimo Venturelli, Carlo Zancanaro, Chiara Milanese

**Affiliations:** Department of Neurosciences, Biomedicine and Movement Sciences, University of Verona, Verona, Italy

**Keywords:** body composition, skinfold thickness, athletes with physical impairments, predictive equation, bland-altman analysis

## Abstract

To date there is no anthropometric equation specific to athletes with unilateral lower limb amputation to estimate the percentage of fat mass (%FM). This study investigated the accuracy of a set of anthropometric equations validated on able-bodied populations to predict the %FM assessed by-means of dual-energy x-ray absorptiometry (DXA) in athletes with unilateral lower limb amputation. Furthermore, a predictive anthropometric equation specific to athletes with unilateral lower limb amputation was developed from skinfold thickness measurements using DXA as the reference method for the estimation of the %FM. Twenty-nine white male athletes with unilateral lower limb amputation underwent a DXA scan and an anthropometric assessment on the same day. The %FM, calculated through several existing anthropometric equations validated upon able-bodied populations, was compared with the DXA-measured %FM (%FM_DXA). Accuracy and agreement between the two methods was computed with two-tailed paired-sample *t*-test, concordance correlation coefficient, reduced major axis regression and Bland-Altman analysis. A stepwise multiple regression analysis with the %FM_DXA as the dependent variable and age and nine skinfold thicknesses as potential predictors was carried out and validated using a repeated 10-fold cross-validation. A linear regression analysis with the sum of nine skinfolds as the independent variable was also carried out and validated using a repeated 10-fold cross-validation. The results showed that the anthropometric equations validated on able-bodied populations are inaccurate in the estimation of %FM_DXA with an average bias ranging from 0.51 to −13.70%. Proportional bias was also found revealing that most of the anthropometric equations considered, tended to underestimate/overestimate the %FM_DXA as body fat increased. Regression analysis produced two statistically significant models (*P* < 0.001 for both) which were able to predict more than 93% of total variance of %FM_DXA from the values of four skinfold measurements (i.e., thigh, abdominal, subscapular and axillary skinfold measurements) or from the sum of 9 skinfolds. Repeated cross-validation analysis highlighted a good predictive performance of the proposed equations. The predictive equations proposed in this study represent a useful tool for clinicians, nutritionists, and physical conditioners to evaluate the physical and nutritional status of athletes with unilateral lower limb amputation directly in the field.

## Introduction

Amputation is defined as the total or partial absence of bones and/or joints as a result of trauma, illness (e.g., bone cancer or diabetes) or congenital anomalies (Simim et al., 2013). Among the various types of physical impairments, amputation on a lower limb has been shown to have a high prevalence worldwide (Wasser et al., 2020). Today, people suffering from unilateral lower limb amputation are eligible to participate in a wide range of adapted sports, such as amputee soccer, wheelchair basketball, wheelchair tennis, track and field, para swimming, handbike and so on (World Amputee Football Federation, 2005; International Paralympic Committee, 2019). In recent years, participation in such adapted sports is constantly growing around the world (Ozkan et al., 2012; Simim et al., 2013; International Paralympic Committee, 2017, 2019).

After a lower-limb amputation, subjects undergo changes in their body composition including increased whole-body adiposity (Sherk et al., 2010) along with muscle atrophy and an increase in the amount of fat mass in the residual limb (Sherk et al., 2010). Such changes in body composition are associated with negative consequences from both a health (Anderson et al., 2013) and a performance perspective (Ozkan et al., 2012). Accordingly, an accurate assessment of body composition in athletes with unilateral lower limb amputation is of great importance in view of assessing their nutritional and training status, as well as monitoring the impact of dietary and training interventions.

Today, the Dual-Energy X-Ray Absorptiometry (DXA) is recognized as an accurate method to objectively assess body composition in athletes with a physical impairment (Keil et al., 2014). However, DXA may not be readily available in many clinical and sport settings due to logistics and costs. As an alternative, in sport practice as well as in several scientific studies (Iturricastillo et al., 2015; Cavedon et al., 2018a, 2015), anthropometry is often employed as a cost-effective and accessible method to assess body composition directly in the field.

The accuracy of anthropometry lies in the use of predictive equations which are specific to the population under evaluation (Heyward and Wagner, 2004; Reilly et al., 2009). In fact, predictive equations are based on the assumption that within each population body fat is distributed subcutaneously and internally in a similar manner in all individuals (Heyward and Wagner, 2004). Body fat distribution is influenced by several factors and varies for example across age groups, gender, ethnicity, and, in the case of athletes, also the particular sport practiced (Reilly et al., 2009; Cavedon et al., 2018b). Furthermore, in athletes with a physical impairment, some studies (Willems et al., 2015; Goosey-Tolfrey et al., 2016; Flueck, 2020) has underlined that the distribution of fat mass is also influenced by the type of physical impairment (e.g., spinal cord injury or lower limb amputations), and the associated modality of daily ambulation (i.e., wheelchair, prosthesis or crutches).

Many predictive anthropometric equations are nowadays available for different populations of able-bodied subjects, both non-athletes (Katch and McArdle, 1973; Durnin and Womersley, 1974; Pollock et al., 1976; Ball et al., 2004; Eston et al., 2005; O’Connor et al., 2010; Bacchi et al., 2017) and athletes (White et al., 1980; Thorland et al., 1984; Evans et al., 2005; Reilly et al., 2009; Oliver et al., 2012; Cavedon et al., 2018b).

To the best of our knowledge, no anthropometric equation specific to athletes with unilateral lower limb amputation has been developed yet. Moreover, the capability of the anthropometric equations developed in able-bodied populations in predicting the DXA-measured whole-body percentage of fat mass (%FM_DXA_) has never been investigated in athletes with unilateral lower limb amputation. Some studies showed a lack of transferability of the anthropometric equations developed for able-bodied populations (Wilmore and Behnke, 1970; Durnin and Womersley, 1974) to athletes with a physical impairment (e.g., athletes with spinal cord injury or mixed groups of athletes with different types of physical impairments) (Sutton et al., 2009; Willems et al., 2015; Goosey-Tolfrey et al., 2016). Taken together, these studies (Goosey-Tolfrey et al., 2016; Sutton et al., 2009; Willems et al., 2015) revealed that such anthropometric equations systematically underestimate the %FM_DXA_ in athletes with a physical impairment.

The aim of the present study was to investigate the ability of a set of anthropometric equations validated in able-bodied athletic and non-athletic populations to estimate the %FM_DXA_ in athletes with unilateral lower limb amputation. Furthermore, population-specific predictive equations for %FM were developed from skinfold thickness measurements using DXA as the reference method.

## Materials and Methods

### Participants

The required sample was estimated “*a priori*” using G^∗^Power ver.3.1.9.2 (Faul, 2009). Setting the type I error at α = 0.05 and the effect size at *d* = 0.60, the minimum sample size required for the two-tailed paired-sample *t*-test to reach an 80% power (i.e., β = 0.20) was 24 subjects. In order to comply with a possible ∼20% dropout, thirty participants were initially recruited. Inclusion criteria were practicing an adapted sport at a competitive level for more than 1 year prior to testing and not suffering from any chronic or systemic disease or other physical impairments, apart from the amputation, that might affect body composition. One athlete did not complete the measurements therefore twenty-nine male white athletes with unilateral lower limb amputation were considered for the analysis. These athletes suffered from amputation through the hip or transfemoral amputation (*n* = 14), amputation through the knee or transtibial amputation (*n* = 13), amelia of a lower limb (*n* = 1), leg length discrepancy (*n* = 1). The mean age of the athletes was 35.86 ± 9.11 years. The cause of amputation was traumatic in 27 athletes and congenital in 2 athletes. In the case of amputation due to a trauma, the duration of injury was 13.8 ± 1.9 years. Athletes had been regularly involved in amputee soccer (*n* = 11), sitting volley (*n* = 1), wheelchair basketball (*n* = 4), track and field (*n* = 4), paratriathlon (*n* = 2), para ice hockey (*n* = 1), skydiving (*n* = 1), and handbike (*n* = 5) for 7.5 ± 1.2 years. All athletes competed at national level and 15 of them competed also at international level. The weekly amount of training was 5.0 ± 0.4.

When assessing the possible impact of the level of amputation on the %FM_DXA_ and the DXA-measured regional %FM, athletes were divided into two groups: athletes with above-knee amputation (AKA, *n* = 16, including athletes with transfemoral lower limb amputation and athletes with amelia) and athletes with below-knee amputation (BKA, *n* = 14, including athletes with transtibial lower limb amputation and athletes with leg length discrepancy).

The study was conducted in accordance with the Declaration of Helsinki, and the protocol was approved by the Institutional Review Board of the local University. All the participants were informed about the aims of the study and the experimental procedures, and they knew that they could withdraw at any time. All participants read and signed the informed consent form.

### Procedures

On the same day, athletes underwent an anthropometric evaluation and a whole-body DXA scan. The measurement session took place in the morning, after a 3–4 h fast. During all measurements the participants wore minimal clothing (i.e., underwear). All participants were asked not to undertake any strenuous physical activity the day before each measurement session and they were also required not to undertake any exercise on the morning of the measurements.

The participants were asked to wear their prosthesis before having their body weight and stature measured. Body mass was assessed with prosthesis to the nearest 0.1 kg using a certified electronic scale (Tanita electronic scale BWB-800 MA, Wunder SA.BI. Srl, Milan, Italy). The weight of the prosthesis was then taken and subtracted from the previous weight with the prosthesis to get the actual body mass. Standing height was measured to the nearest 0.1 cm using a Harpenden portable stadiometer (Holtain Ltd., Crymych, Pembs. United Kingdom) according to conventional criteria and measuring procedures (Lohman et al., 1988).

Body composition was assessed by means of Dual-Energy X-ray Absorptiometry (DXA) on a QDR Explorer fan beam densitometer (Hologic, MA, United States). In our laboratory quality control of the DXA scanner is performed daily before actual use by means of an encapsulated spine phantom (Hologic Inc., Bedford, MA, United States) to check for possible baseline drift. Participants undertook whole-body DXA scanning according to “The Best Practice Protocol for the assessment of whole-body body composition by DXA” (Nana et al., 2015). Positioning aids to support the residual lower limb were employed and special strapping was applied around participants’ residual ankle to ensure there was no movement during the scans. Prior to scanning, participants were asked to void their bladder and to remove all metal, jewelry or reflective material, including prostheses.

Analysis of DXA scans was performed using Hologic Discovery software for Windows XP version 12.6.1 according to the manufacturer’s procedures to get the %FM_DXA_ and the percentage of fat mass (%FM) in the trunk, arms (right and left) and legs (right and left) regions. For analysis at the regional level, the average %FM of the right and the left arm (arms) and the %FM of the non-impaired leg were used. The same trained investigator carried out all measurements and analyzed all the DXA scans to ensure consistency.

Skinfold thicknesses were measured to the nearest 0.2 mm by the same trained investigator with a Harpenden caliper (Gima, Milan, Italy) at the biceps, triceps, subscapular, chest, axilla, suprailiac, abdominal, anterior thigh (of the non-impaired leg) and calf (of the non-impaired leg) according to standard procedures (Lohman et al., 1988). Duplicate readings were taken at each site, and the average of the two was recorded.

Body density or the %FM were calculated using nine anthropometric equations developed in able-bodied athletic populations (Forsyth and Sinning, 1973; White et al., 1980; Thorland et al., 1984; Evans et al., 2005; Reilly et al., 2009; Oliver et al., 2012) and nine anthropometric equations developed in able-bodied non-athletic populations (Nagamine and Suzuki, 1964; Sloan, 1967; Wilmore and Behnke, 1970; Katch and McArdle, 1973; Durnin and Womersley, 1974; Pollock et al., 1976; Ball et al., 2004; Eston et al., 2005; O’Connor et al., 2010; Table 1). Body density values were converted to %FM according to Siri (1961).

**TABLE 1:** Anthropometric equations used to predict the body density or the percentage of body fat in athletes with unilateral lower limb amputation.

References	Abbreviation	Anthropometric equation
Forsyth and Sinning, 1973	Eq_Fs2	BD = 1.1103 – (0.00168 ⋅ SS) – (0.00127 ⋅ AB)
Forsyth and Sinning, 1973	Eq_Fs4	BD = 1.10647 – (0.00162 ⋅ SS) – (0.00144 ⋅ AB) – (0.00077 ⋅ TR) + (0.00071 ⋅ AX)
Thorland et al., 1984	Eq_Th3	BD = 1.1136 – [0.00154 ⋅ (TR + SS + AX)] + [0.00000516 ⋅ (TR + SS + AX)^2^]
Thorland et al., 1984	Eq_Th7	BD = 1.1091 – [0.00052 ⋅ (TR + SS + AX + SI + AB + TH + CA)] + + [0.00000032 ⋅ (TR + SS + AX + SI + AB + TH + CA)^2^]
White et al., 1980	Eq_Whi	BD = 1.0958 – (0.00088 ⋅ SI) – (0.0006 ⋅ TH)
Evans et al., 2005	Eq_Ev3	%FM = 8.997 + [0.24658 ⋅ (TR + SI + TH)] – (6.343 ⋅ Sex) – (1.998 ⋅ Race)
Evans et al., 2005	Eq_Ev7	%FM = 10.566 + [0.12077 ⋅ (TR + SI + TH + SS + AX + CH + AB)] – (8.057 ⋅ Sex) – (2.545 ⋅ Race)
Oliver et al., 2012	Eq_Oli	%FM = 3.53 + [0.132 ⋅ (TR + SI + TH + SS + AX + CH + AB)]
Reilly et al., 2009	Eq_Rei	%FM = 5.174 + (0.124 ⋅ CH) + (0.147 ⋅ AB) + (0.196 ⋅ TR) + (0.13 ⋅ CA)
Durnin and Womersley, 1974	Eq_DWg	BD = 1.1765 – [0.0744 ⋅ log_10_ (BI + TR + SS + SI)]
Katch and McArdle, 1973	Eq_KMc	BD = 1.09665 – (0.00103 ⋅ TR) – (0.00056 ⋅SS) – (0.00054 ⋅ AB)
Nagamine and Suzuki, 1964	Eq_NaS	BD = 1.0913 – [0.00116 ⋅ (TR + SS)]
Pollock et al., 1976	Eq_Pol	BD = 1.09716 – (0.00065 ⋅ CH) – (0.00055 ⋅ SS) – (0.0008 ⋅TH)
Sloan, 1967	Eq_Slo	BD = 1.1043 – (0.001327 ⋅ TH) – (0.00131 ⋅SS)
Wilmore and Behnke, 1970	Eq_WiB	BD = 1.08543 – (0.000886 ⋅ AB) – (0.0004 ⋅ TH)
Ball et al., 2004	Eq_Bal	%FM = 0.465 + [0.18 ⋅ (CH + AX + TR + SS + AB + SI + TH)] – – [0.0002406 ⋅ (CH + AX + TR + SS + AB + SI + TH)^2^] + (0.06619 ⋅ age)
Eston et al., 2005	Eq_Est	%FM = [0.12 ⋅ (BI + TR + SS + SI)] + [0.36 ⋅ (TH + CA)] + 1.61
O’Connor et al., 2010	Eq_Oco	%FM = [0.272 ⋅ (TR + SI + TH)] – [0.0005 ⋅ (TR + SI + TH)] + 4.972

*BD, body density; %FM, percentage of body fat; SS, subscapular; AB, abdominal; TR, triceps; AX, axilla; SI, suprailiac; TH, thigh; CA, calf; CH, chest; BI, biceps; Sex, male = 1 and female = 0; Race, black = 1 and white = 0.*

### Statistical Analysis

The normality of data was checked, and all variables did not show significant deviations from the gaussian distribution. Descriptive statistics (mean and standard deviation) were computed for all variables.

Differences in the %FM_DXA_ and the DXA-measured regional %FM between the AKA e BKA groups were assessed by the two-tailed independent-sample *t* test. As the AKA and BKA groups were similar in both the %FM_DXA_ and the regional %FM, the level of amputation was not taken into account for further analyses and the study sample was considered as a whole.

Mean bias (i.e., the average of the differences between the %FM obtained by each anthropometric equation and the %FM_DXA_) was computed to get a measure of systematic measurement errors. The %FM obtained by each anthropometric equation was compared with the %FM_DXA_ using the two-tailed paired-sample *t*-test. The Lin’s concordance correlation coefficient (ρc) was used to quantify the agreement between the %FM_DXA_ and each anthropometric equation (Lin, 1989); agreement was considered poor (ρc < 0.90), moderate (ρc between 0.90 and 0.95), substantial (ρc between 0.95 and 0.99), excellent (ρc > 0.99) and perfect (ρc = 1). The intraclass correlation coefficient (r) was also calculated as a measure of validity according to Fisher (Fisher, 1950; page 212) and interpreted according to Cicchetti (1994) as poor (*r* < 0.40), fair (r between 0.40 and 0.59), good (r between 0.60 and 0.74) and excellent (r between 0.75 and 1.00). The Reduced Major Axis (RMA) regression (Harper, 2014) was used to assess the relationship between the %FM_DXA_ (i.e., the dependent variable) and the %FM predicted by each anthropometric equation. In case of perfect agreement, the intercept and the slope of the RMA line are 0 and 1, respectively.

Agreement between each anthropometric equation and DXA was tested using Bland-Altman analysis (limits of agreement and range) (Bland and Altman, 2012). The presence of proportional bias was explored by examining the association between the mean bias and the average of the two methods (i.e., the average between the %FM obtained with each anthropometric equation and the %FM_DXA_) by the Pearson’s product-moment correlation coefficient (r). The strength of the correlation coefficient was considered small (*r* = 0.00–0.30), moderate (*r* = 0.31–0.49), large (*r* = 0.50–0.69), very large (*r* = 0.70–0.89), and almost perfect for assessing relationships (*r* = 0.90–1.00) as suggested by Hopkins (2016).

A stepwise multiple regression analysis was carried out with the %FM_DXA_ as the dependent variable and with age and 9 skinfold thicknesses (i.e., biceps, triceps, subscapular, axilla, chest, suprailiac, abdominal, anterior thigh, and calf skinfolds) as potential predictors. The value of probability of F-to-enter was equal to 0.05 and the probability of F-to-remove was equal to 0.10. The adjusted coefficient of determination (R^2^) and the standard error of estimate (SEE) were used to represent the goodness-of-fit of the predictor model. Homoscedasticity of data was assessed by plotting the residuals of multiple regression analysis against the predicted values. The Durbin-Watson test was used to test for autocorrelation in the residuals, while the variance inflation factor and the condition index were calculated to test collinearity. A linear regression analysis with the %FM_DXA_ as the dependent variable and the sum of 9 skinfolds as the independent variable was also carried out. For both the regression models, Cohen’s *f* squared (*f*^2^) was used to calculate the effect size of the regression model and interpreted as small (*f*^2^ ≥ 0.02), medium (*f*^2^ ≥ 0.15) and large (*f*^2^ ≥ 0.35) according to Cohen’s guidelines (Cohen, 1988). The developed regression models were then validated using a repeated 10-fold cross-validation (with 1000 replications), estimating for each cross-validation sample the root mean squared prediction error (RMSPE), the coefficient of determination R^2^, and the mean absolute prediction error (MAPE).

Statistical analyses were performed using SPSS v. 16.0 (IBM Corp., Armonk, NY, United States) and R-4.0.3 (Foundation for Statistical Computing, Vienna). The statistical significance was set at *P* ≤ 0.05.

## Results

Descriptive statistics (mean and standard deviation) for age, %FM_DXA_ and the DXA-measured regional %FM of the whole sample as well as for the AKA and BKA groups are reported in Table 2. The *t*-test showed no statistically significant differences between the AKA and BKA groups in age, %FM_DXA_ and the DXA-measured regional %FM (Table 2).

**TABLE 2:** Participant’s age and DXA-measured whole-body and regional percentage of fat mass.

	Whole sample (*n* = 29)		AKA (*n* = 15)		BKA (*n* = 14)		Independent-sample *t*-test	
Variables	Mean	SD	Mean	SD	Mean	SD	*t*	*P*
Age (years)	35.86	9.11	35.22	12.91	37.08	13.48	0.440	0.664
%FM_DXA_	21.67	4.40	21.80	4.05	22.20	4.23	0.147	0.884
Trunk%FM	21.16	5.39	21.04	5.33	22.11	5.56	0.608	0.549
Arms%FM	19.42	4.51	19.16	4.82	20.28	5.03	0.417	0.680
N-I leg%FM	22.44	4.80	22.98	4.99	22.50	5.21	–0.318	0.753

*AKA, above-knee amputee athletes; BKA, below-knee amputee athletes; SD, standard deviation; %FM_DXA_, DXA-measured whole-body percentage of fat mass; %FM, percentage of fat mass; N-I, non-impaired.*

A summary of the results related to accuracy and agreement in the estimate of the %FM by each anthropometric equation versus the %FM_DXA_ is presented in Table 3. The *t*-test showed that only the average %FM obtained with the Eq_DWg (Durnin and Womersley, 1974) was similar to the average %FM_DXA_, while all other anthropometric equations were significantly different to the %FM_DXA_. The limits of agreement showed that 95% of the time, the Eq_DWg (Durnin and Womersley, 1974) produced %FM estimates that were between −3.8% less and 4.9% higher than the %FM_DXA_. The two-tailed paired-sample *t*-test also revealed that the Eq_Fs4 (Forsyth and Sinning, 1973) significantly overestimated the %FM when compared with the %FM_DXA_, with a systematic bias equal to 1.31%. In this anthropometric equation, the 95% limits of agreement of the bias ranged from −2.5% less and to 5.1% more. All other anthropometric equations significantly underestimated the %FM versus %FM_DXA_ (Table 3), with systematic bias ranging from −1.14% (Eq_Bal) to −8.03% (Eq_Whi). The ρc showed poor agreement between the %FM obtained with each of the considered anthropometric equations and the %FM_DXA_ (ρc < 0.90 for all, Table 3). According to Cicchetti (1994), the intraclass correlation coefficient r was considered poor (Eq_Whi, Eq_Ev3; Eq_Ev7; Eq_Rei; Eq_KMc; Eq_NaS; Eq_Pol), fair (Eq_Th3, Eq_Slo; Eq_WiB), good (Eq_Oli, Eq_Est, Eq_Oco) or excellent (Eq_Fs2, Eq_Fs4, Eq_Th7, Eq_DWg, Eq_Bal). The slope and the intercept of the RMA were, respectively, near to 1 and to 0 for the %FM_DXA_ and the Eq_Fs2 as well as for the %FM_DXA_ and the Eq_Est indicating good concordance between these measurements. All other measurements showed poor agreement.

**TABLE 3:** Analysis of the agreement between the percentage of fat mass obtained with anthropometric equations and the percentage of fat mass obtained by-means of DXA.

	Descriptive statistics		Paired *t*-test			CCC	ICC	RMA regression		Bland-Altman analysis			
AE	Mean	SD	Bias	t	P	ρc	r	Slope	Int	U LoA	L LoA	Range	r
Eq_Fs2	20.16	4.15	−1.51	5.45	<0.001	0.88	0.88	0.94	−0.23	1.42	−4.45	5.86	−0.18
Eq_Fs4	22.98	4.28	1.31	−3.69	0.001	0.86	0.86	0.97	1.95	5.06	−2.45	7.50	−0.07
Eq_Th3	17.73	4.01	−3.94	11.00	<0.001	0.62	0.55	0.91	−1.96	−0.16	−7.72	7.56	−0.21
Eq_Th7	19.91	4.48	−1.76	7.214	<0.001	0.88	0.88	1.02	−2.08	0.82	−4.34	5.16	0.05
Eq_Whi	13.64	3.20	−8.03	20.70	<0.001	0.26	0.13	0.73	−2.08	−3.93	−12.12	8.19	−0.59
Eq_Ev3	14.94	2.91	−6.73	17.53	<0.001	0.32	0.00	0.66	0.63	−2.66	−10.80	8.13	−0.74
Eq_Ev7	16.68	2.83	−4.98	14.94	<0.001	0.46	0.28	0.64	2.76	−1.45	−8.53	7.08	−0.88
Eq_Oli	19.02	3.10	−2.64	8.93	<0.001	0.73	0.70	0.70	3.81	0.49	−5.79	6.28	−0.83
Eq_Rei	14.25	1.52	−7.42	13.33	<0.001	0.16	0.31	0.35	6.76	−1.53	−13.31	11.78	−0.97
Eq_DWg	22.18	3.25	0.51	−1.25	0.221	0.83	0.83	0.74	6.22	4.86	−3.84	8.71	−0.54
Eq_KMc	15.96	2.48	−5.70	13.32	<0.001	0.34	0.08	0.56	3.77	−1.17	−10.24	9.07	−0.85
Eq_NaS	17.51	2.70	−4.16	8.47	<0.001	0.44	0.30	0.61	4.26	1.03	−9.35	10.39	−0.68
Eq_Pol	14.88	3.29	−6.78	19.29	<0.001	0.34	0.05	0.75	−1.27	−3.06	−10.51	7.46	−0.60
Eq_Slo	17.51	4.75	−4.16	9.60	<0.001	0.61	0.54	1.08	−5.85	0.41	−8.74	9.16	0.15
Eq_WiB	18.43	2.42	−3.24	7.31	<0.001	0.54	0.46	0.55	6.53	1.45	−7.94	9.39	−0.85
Eq_Bal	20.52	3.29	−1.14	3.61	0.001	0.86	0.86	0.75	4.36	2.22	−4.51	6.73	−0.66
Eq_Est	18.90	3.86	−2.77	7.09	<0.001	0.71	0.68	0.88	−0.07	1.36	−6.90	8.25	−0.27
Eq_Oco	18.50	3.21	−3.17	8.88	<0.001	0.65	0.60	0.73	2.75	0.61	−6.95	7.56	−0.64

*CCC, concordance correlation coefficient; ICC, intraclass correlation coefficient r; RMA, reduced major axis; AE, anthropometric equations; SE, standard error; P, *P*-value; U, upper; L, lower; LoA, limits of agreement; r, Pearson’s correlation coefficient indicating the association between the difference and the mean between the two methods; ρc, Lin’s concordance correlation coefficient; Int, intercept.*

**FIGURE 1:**
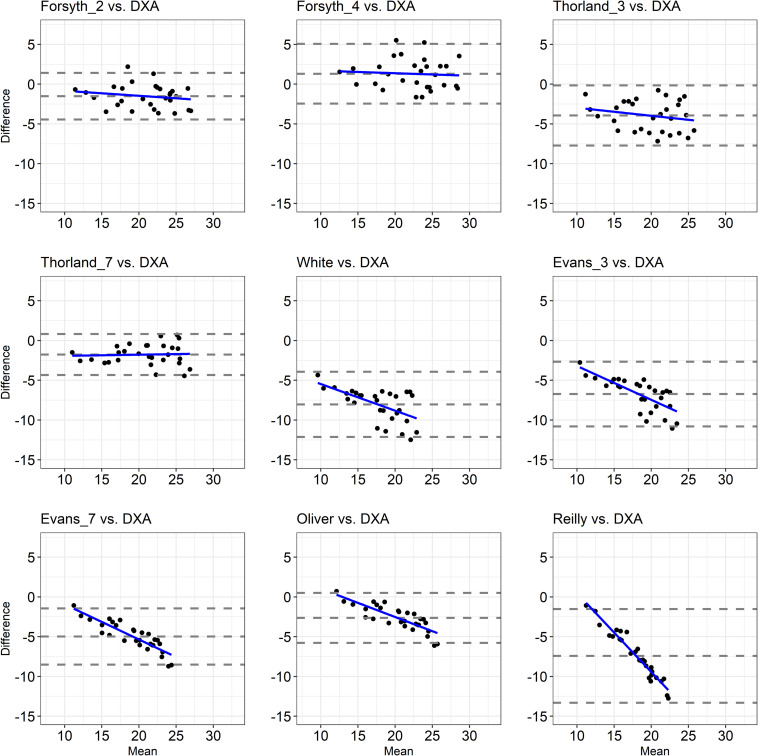
Bland-Altman Plots showing the agreement between the %FM assessed through anthropometric equations validated upon able-bodied athletic populations and the %FM_DXA_. The dashed lines indicate bias ± 2 standard deviations and the solid blue line indicates the regression line.

**FIGURE 2:**
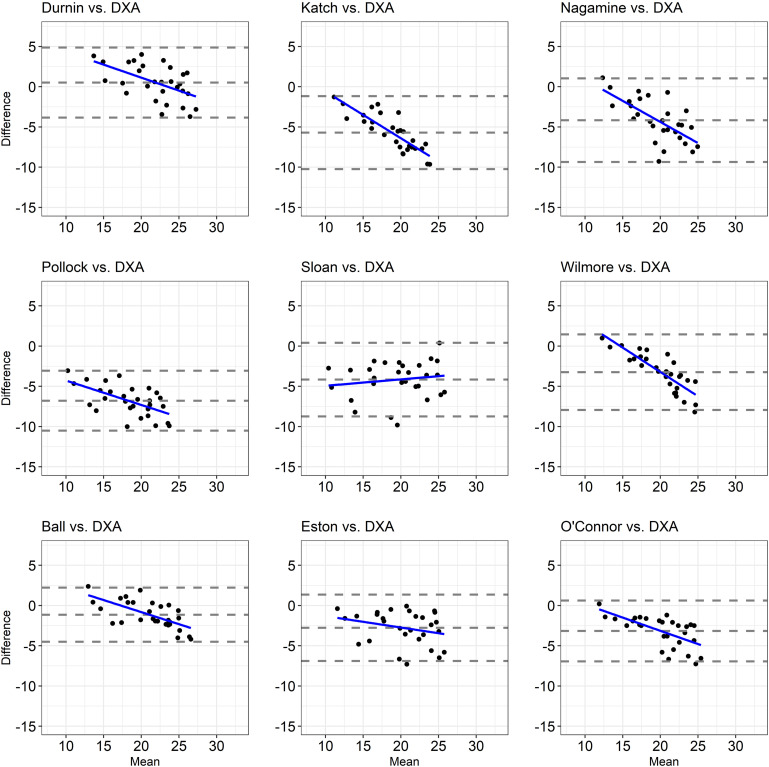
Bland-Altman Plots showing the agreement between the %FM assessed through anthropometric equations validated upon able-bodied non-athletic populations and the %FM_DXA_. The dashed lines indicate bias ± 2 standard deviations and the solid blue line indicates the regression line.

The agreement between the %FM obtained by a set of anthropometric equations and the %FM_DXA_ is depicted in Figures 1, 2. Despite at least 93% of the data points falling within the 95% limits of agreement in each Bland-Altman plot (Figures 1, 2), the range of the limits of agreement in each plot was large (i.e., higher than 5.16; Table 3), indicating poor agreement.

The correlation analysis showed that the association between the difference and the mean values of the two methods used to estimate the %FM (i.e., each anthropometric equation and DXA) was small (Eq_Fs2 vs. DXA; Eq_Fs4 vs. DXA; Eq_Th3 vs. DXA, Eq_ Th7 vs. DXA, Eq_Slo vs. DXA, Eq_Est vs. DXA), large (Eq_Whi vs. DXA, Eq_DWg vs. DXA, Eq_Nas vs. DXA, Eq_Pol vs. DXA, Eq_Bal vs. DXA, Eq_Oco vs. DXA), very large (Eq_Ev3 vs. DXA, Eq_Ev7 vs. DXA, Eq_Oli vs. DXA, Eq_KMc vs. DXA, Eq_WiB vs. DXA) and almost perfect (Eq_Rei vs. DXA), indicating that as the %FM_DXA_ increased, so did the underestimation/overestimation of the %FM with most of the considered anthropometric equations (Table 3 and Figures 1, 2).

In the whole sample, by entering age and nine skinfold thicknesses (i.e., biceps, triceps, subscapular, axilla, chest, suprailiac, abdominal, anterior thigh, and calf skinfolds) as potential predictors in stepwise multiple regression analysis, a statistically significant model was estimated for %FM_DXA_ (*F* = 116.272, *P* < 0.001). The model was:

$$\begin{array}{c} \%FM_{DXA}=0.241\;(Anterior\;Thigh\;Skinfold)\;+\;0.418\\ \left(Abdominal\;Skinfold)\;+\;0.329\;(Subscapular\;Skinfold\right)\\ +\;0.259\;(Axillary\;Skinfold)\;-1.689. \end{array}$$

Adjusted R^2^, SEE and *f*^2^ were 0.943, 1.05 and 16.54, respectively. The Durbin-Watson was 2.30, indicating that there was no autocorrelation between the residuals. The variance inflation factor and the condition index were, respectively, < 2.6 and < 24.9 for all the predicting variables showing that multicollinearity between the variables in the model was weak.

The validation of our model using a repeated 10-fold cross-validation, showed that the model has good predictive performance (mean RMSPE = 1.51 [range = 1.06–2.18]; mean *R*^2^ = 0.89 [range = 0.78–0.94]; mean MAPE = 1.18 [range = 0.85 – 1.73]).

As several of the existing anthropometric equations used the sum of skinfolds as a predictor of the body density or the %FM, we also calculated the prediction of a regression model using the sum of 9 skinfolds as the independent variable. Analysis yielded a statistically significant model (*F* = 357.63, *P* < 0.001) with a good predictivity (*R*^2^ = 0.93). The model was:

$$\%FM_{DXA}\;=\;0.162\;(SumofNineSkinfolds)-0.311.$$

SEE and *f*^2^ were 1.19 and 13.08, respectively.

The validation of our model using a repeated 10-fold cross-validation, showed that the model has good predictive performance (mean RMSPE = 1.23 [range = 1.15–1.39]; mean *R*^2^ = 0.92 [range = 0.90–0.93]; mean MAPE = 1.03 [range = 0.96–1.18]).

Bland-Altman plots of the developed equations have been provided as Supplementary Material.

## Discussion

Evaluating and monitoring body composition is a key issue in adapted sports practice due to its link to both health and performance. The accurate assessment of body composition in athletes with a physical impairment is a difficult task due to the different types of physical impairment (e.g., spinal cord injury or limb amputation/s) and the consequent differences in the distribution of body fat in comparison with able-bodied persons (Willems et al., 2015). To the best of our knowledge, there is no scientific data related to the capability of field measurements (e.g., skinfold thickness) in accurately assessing body composition in athletes with unilateral lower limb amputation. In order to fill this gap in the scientific literature, this study aimed to assess the ability of a set of anthropometric equations validated upon athletic and non-athletic able-bodied populations in estimating the %FM in athletes with unilateral lower limb amputation, using the %FM_DXA_ as the criterion. Furthermore, this study is the first attempt to develop anthropometric equations specific for this athletic population to predict the %FM_DXA_.

A primary finding of the present study was that almost all of the anthropometric equations obtained from both athletic and non-athletic able-bodied populations are inaccurate in estimating the %FM_DXA_ in athletes with unilateral lower limb amputation. The %FM obtained by the Eq_DWg was the only anthropometric equation able to predict the %FM close to the %FM_DXA_ with a mean bias equal to 0.51%, the difference being not statistically significant (Table 3). However, the Bland-Altman plot (Figure 2) showed that, even if all points fell within the 95% limits of agreement, the range of the limits of agreement was wide (i.e., 8.71%) making the prediction inaccurate. For all the other anthropometric equations under investigation, the two-tailed paired-sample *t*-test revealed a statistically significant, systematic bias leading to underestimation or overestimation of %FM_DXA_ in athletes with unilateral lower limb amputation (Table 3). More specifically, of the nine anthropometric equations validated in athletic able-bodied populations, only one equation (i.e., Eq_Sf4) overestimated the %FM_DXA_, while the other eight equations underestimated the %FM_DXA_. Instead, all anthropometric equations validated in non-athletic able-bodied populations (except Eq_DWg) systematically underestimated the %FM_DXA_. Moreover, a large (> ± 4%) systematic bias was found in 8 out of 18 of the evaluated anthropometric equations (4 validated upon athletes and 4 validated upon non-athletes) (Table 3).

These results are partially in line with data from others (Willems et al., 2015) reporting that four anthropometric equations validated in non-athletic healthy populations (Sloan and Weir, 1970; Durnin and Womersley, 1974; Lean et al., 1996; Gallagher et al., 2000; Pongchaiyakul et al., 2005) systematically underestimated the %FM_DXA_ with a mean bias ranging from −2.1% in the equation by Sloan and Weir to −9.0% in the equation of Lean et al. It is important to underline that the study sample of Willems and colleagues (Willems et al., 2015) was composed of seven wheelchair-game athletes who were “wheelchair independent during non-sport activities”; five of them had unilateral lower limb amputation and two had lower limb deficiencies. It is interesting to note that the mean bias found in the present study for the equation by Durnin and Womersley (0.51%, non-significant) was lower than that found in the study by Willems and colleagues (−4.2%, statistically significant). This may be due to the fact that our study sample was homogeneous for the type of physical impairment (i.e., all athletes had unilateral lower limb amputation). Accordingly, it can be supposed that, in athletes with unilateral lower limb amputation only, the Durnin and Womersley equation may be more accurate in predicting the %FM_DXA_ than in athletes with different types of physical impairment.

Proportional bias in most comparisons between the %FM_DXA_ and the considered anthropometric equations suggests that the accuracy of the anthropometric equations is affected by the value of %FM_DXA_. Accordingly, anthropometric equations are more inaccurate in athletes with higher values of %FM_DXA_. It is important to take in mind that athletes in this study had values of %FM_DXA_ ranging from 11.8 to 28.7%. This wide range of %FM_DXA_ values may be due to the fact that athletes competed in different adapted sports and their weekly amount of training for athletes ranges from 4 to 12 h. Future research aimed at investigating the transferability of anthropometric equations, validated upon able-bodied populations, should also split up athletes according to the type of adapted sport they are practicing (e.g., endurance or team sports) and possibly also their range of %FM, for example grouping athletes with a %FM lower than 15%, between 15 and 20%, between 20 and 25%, greater than 25%.

Taken together these results suggest that in athletes with unilateral lower limb amputation, the anthropometric equations validated upon able-bodied populations are inadequate in accurately predicting the %FM_DXA_, regardless of whether they have been validated in athletes or in non-athletes. Accordingly, these results underline the need for anthropometric equations for estimating the %FM in this specific athletic population.

This study represents the first attempt to develop population-specific anthropometric equations to predict the %FM_DXA_ in athletes with unilateral lower limb amputation. The developed equations using four skinfold thicknesses (i.e., anterior thigh, abdominal, subscapular, and axillary) and the sum of 9 skinfolds as predictors are able to predict more than 93% of total variance in %FM_DXA_ with a small SEE (about 1% for both) and a large effect size (*f*^2^ > 13.1). Moreover, repeated cross-validation analysis highlighted a good predictive performance of both proposed equations. Accordingly, the anthropometric equations developed in the current study accurately estimate the %FM_DXA_ and can therefore be used as a field tool to assess the %FM in white male adult athletes with unilateral lower limb amputation. Moreover, they may even represent a useful tool for clinicians, nutritionist, sport doctors and physical conditioners to get important information about the nutritional status of their athletes.

This study has some limits to be mentioned. A first limitation is the small sample size. However, it is important to consider that this study deals with a highly specific population (i.e., athletes with unilateral lower limb amputation) making it difficult to recruit a large number of participants especially when they are required to travel in order to reach the site of data collection (e.g., to undergo laboratory tests). A second limit of the study was the limited number of measured anthropometric variables, which prevented the use of some anthropometric equations which could be applied in this population (e.g., Kanellakis et al., 2017). Third, the provided anthropometric equations were only cross-validated. Accordingly, future research is needed to validate the anthropometric equation developed in this study in independent samples of athletes with unilateral lower limb amputation. Fourth, we did not consider the type of sport practiced by athletes. Future research is advocated to provide sport-specific anthropometric equations for the different populations of athletes with unilateral lower limb amputation.

In this study there are also some important strengths to underline. First, we used DXA as the reference technique to provide an accurate measure of %FM. DXA measurements are greatly reproducible and the validity of this method has been previously demonstrated also in athletes with a physical impairment (Keil et al., 2014). Second, this is the first study with such a relatively large number of athletes (*n* = 29) with the same type and severity of physical impairment (i.e., unilateral lower limb amputation) and homogeneous for gender, age group and race. Third, in this study we assessed the ability of a large number (*n* = 18) of anthropometric equations in predicting the %FM_DXA_, which had been validated upon both athletes and non-athletes.

In conclusion, this study filled a knowledge gap in the literature by showing that the available anthropometric equations derived from able-bodied populations (both athletic and non-athletic) are inaccurate in athletes with unilateral lower limb amputation in predicting the %FM_DXA_. This further underlines the need for impairment-specific anthropometric equations to estimate body composition in athletes with a physical impairment. As a first step toward this aim, this study produced two anthropometric equations based on skinfold thickness measurements to estimate the %FM_DXA_ in athletes with unilateral lower limb amputation. Nutritionists, clinicians and sports professionals will therefore benefit from using these proposed predictive equations as a rapid, non-invasive tool for assessing and monitoring body composition in athletes with unilateral lower limb amputation.

## Data Availability Statement

The raw data supporting the conclusions of this article will be made available by the authors, without undue reservation.

## Ethics Statement

The studies involving human participants were reviewed and approved by the Institutional Review Board of the University of Verona. The patients/participants provided their written informed consent to participate in this study.

## Author Contributions

VC and CM conceived and designed the experiments. VC performed the experiments. MS and VC analyzed the data and conducted formal analysis. VC and CM wrote the manuscript with support from CZ. MV, CZ, and CM supervised and validated. All authors provided critical feedback and helped shape the research, analysis and manuscript.

## Conflict of Interest

The authors declare that the research was conducted in the absence of any commercial or financial relationships that could be construed as a potential conflict of interest.
