# Supplementary material for: Anthropometric Prediction of DXA-Measured Percentage of Fat Mass in Athletes With Unilateral Lower Limb Amputation
Source: Front Physiol. 2020 Dec 23;11:620040. doi: 10.3389/fphys.2020.620040 (PMC7786292; doi:10.3389/fphys.2020.620040)
Supplement: Supplementary file 1 [file Data_Sheet_1.PDF]

Table 1S. Descriptive statistic (mean and standard deviation) of skinfold thickness measurements.

| **Skinfold thickness** | **Mean** | **Standard deviation** | **Unadjusted $R^2$** |
|---|---|---|---|
| Bicipital (mm) | 6.60 | 1.26 | 0.65 |
| Tricipital (mm) | 12.01 | 2.36 | 0.62 |
| Subscapular (mm) | 15.98 | 3.35 | 0.57 |
| Suprailiac (mm) | 19.38 | 5.43 | 0.65 |
| Axillary (mm) | 13.99 | 3.59 | 0.66 |
| Chest (mm) | 13.53 | 3.73 | 0.59 |
| Abdominal (mm) | 24.04 | 4.38 | 0.67 |
| Thigh (mm) | 18.43 | 5.64 | 0.67 |
| Calf (mm) | 11.60 | 2.39 | 0.42 |
