# Supplementary figures and images for: Anthropometric Prediction of DXA-Measured Percentage of Fat Mass in Athletes With Unilateral Lower Limb Amputation
Source: Front Physiol. 2020 Dec 23;11:620040. doi: 10.3389/fphys.2020.620040 (PMC7786292; doi:10.3389/fphys.2020.620040)

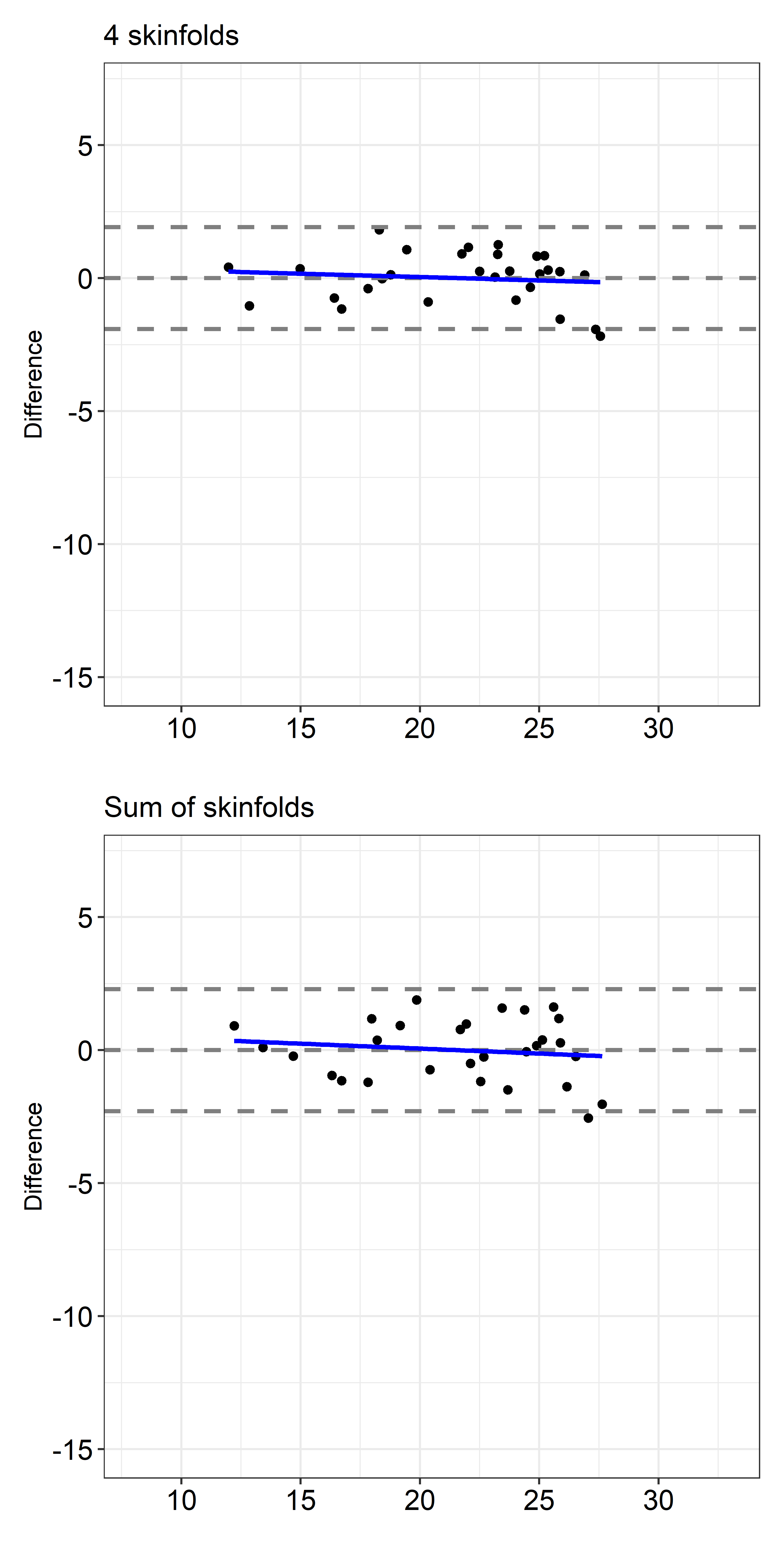

Supplement: Supplementary file 2 [file Image_1.tif]
